# Prevalence of injecting drug use in Estonia 2010–2015: a capture-recapture study

**DOI:** 10.1186/s12954-019-0289-3

**Published:** 2019-03-14

**Authors:** Mait Raag, Sigrid Vorobjov, Anneli Uusküla

**Affiliations:** 1 Institute of Family Medicine and Public Health, University of Tartu, Tartu, Estonia; 2 National Institute for Health Development, Tallinn, Estonia

**Keywords:** Injecting drug use, People who inject drugs, Estonia, Capture-recapture, Over-coverage, Bayesian analysis

## Abstract

**Background:**

It has been observed in an earlier study that the number of people who inject drugs (PWID) in Estonia is declining. We provide nationwide estimates of the number of PWID in Estonia for years 2010–2015 and compare different modelling strategies to minimise over-coverage-induced bias in capture-recapture estimates.

**Methods:**

We obtained data from the Estonian Causes of Death Registry (DR) for opioid-related deaths, the Estonian Health Insurance Fund (HIF) for opioid-related overdose and drug dependence treatment episodes, and the Estonian Police and Border Guard Board (PB) drug-related misdemeanours. Datasets were linked by identifier based on sex, date of birth, and initials; a capture-recapture method was used to estimate the number of PWID aged 15 or more, each year from 2010 to 2015. Log-linear regression maximum likelihood (ML) and Bayesian methods were used; over-coverage of police data was accounted for.

**Results:**

The annual population size estimates of the number of PWID (aged 15 and over) varied from 6000 to 17,300 (ML estimates not accounting for over-coverage of PB) to 1500–2300 (Bayesian estimates accounting for over-coverage). Bayesian estimates indicated a slight decrease in the number of PWID, and the median estimates were > 2000 in years 2010–2012 and < 1800 in years 2013–2015.

**Conclusions:**

Over-coverage of a registry can have a great impact on the estimates of the size of the target population. Bayesian estimates accounting for this over-coverage may provide better estimates of the target population size.

**Electronic supplementary material:**

The online version of this article (10.1186/s12954-019-0289-3) contains supplementary material, which is available to authorized users.

## Background

HIV infection is prevalent in many populations of people who inject drugs (PWID) [1], representing a substantial challenge to public health. Reliable estimates of the size of populations of interest (i.e. PWID) are needed for policy (advocacy, response, planning, resource allocation, and projections of the burden of the disease), programming (intervention planning, measurement of coverage, monitoring and evaluation of interventions), and addressing health inequities. Yet, according to the United Nations Office on Drugs and Crime (UNODC), as of May 2018, there are 120 countries in the world with no estimates on the number of PWID, and 50 countries where the latest estimate on the size of the PWID population is for 2012 or earlier [2].

The capture-recapture method is recommended [3–5] and commonly used (e.g. [6–8]) to estimate the number of PWID, using administrative data sources as capturing samples. These data sources should allow accurate identification of members of the target population. When querying a data source using factors that strongly indicate injecting drug use (e.g. opioid-related overdose in Estonia [9]), the resulting dataset contains PWID almost exclusively. However, some data sources might not record indicators specific to injecting drug use which would allow accurate discrimination of PWID from non-injection drug users or current PWID from former PWID and hence contain a significant number of people who (currently) do not inject drugs (i.e. over-coverage).

Injecting drug use is a driving force behind HIV and HCV epidemics in Eastern Europe and Central Asia [10], including Estonia, where HIV prevalence among people who inject drugs has been estimated at about 50% [11] to 60% [12]. For Estonia, the most recent estimate of the size of the PWID population originates from 2009 [8]. An analysis of changes in the size of PWID population over the period 2004–2009 suggested that the number of PWID in Estonia is decreasing [8]. This finding is in agreement with other studies from Estonia, where it has been observed that the average age of PWID has been increasing (most participants were less than 25 years old in 2005 [13] vs. most were over 30 in 2016 [11]) in parallel with the average number of years they have been injecting (26% up to 3 years in 2005 [14] vs. 12% up to 5 years in 2016 [11]), suggesting that fewer people are starting injecting, potentially leading to a decrease in the number of PWID. Although the demographics of PWID in these studies have changed, the main drug injected by PWID remains a synthetic opioid—fentanyl. In the 2016 study conducted by Uusküla et al., it was found that most PWID (76%) used opioids during the last 6 months (A. Uusküla, unpublished data).

In this paper, we extend the analysis of the number of PWID in Estonia for the years 2010 to 2015 and explore different modelling strategies to minimise over-coverage-induced bias in capture-recapture estimates.

## Methods

### Data

As in the previous study [8], we used four different sets of data covering the years from 2010 to 2015 from three administrative sources, all nationwide, electronic, with no significant changes in data collection or definitions over the period of observation.

The Estonian Causes of Death Registry (DR) was interrogated about deaths related to mental and behavioural disorders due to opioid use (coded as F11.0–F11.9 by the International Classification of Diseases (ICD) v. 10), poisoning by opioids and synthetic narcotics (T40.0–T40.4), accidental poisoning by and exposure to narcotics and psychodysleptics (X42), and intentional self-poisoning by and exposure to narcotics and psychodysleptics (X62). For such deaths, dates of birth and death, sex, initials, and county of residence of the deceased were obtained. In a report covering 9 months in 2012, it was found that 97% of overdose deaths were related to either fentanyl or methadone [15]. We assumed that all deaths in the dataset retrieved from DR were related to injecting drug use.

The Estonian Police and Border Guard Board (PB) provided data on misdemeanours of consumption of narcotic drugs or psychotropic substances without a prescription, or illegal manufacture, acquisition or possession of small quantities of narcotic drugs or psychotropic substances (Act on Narcotic Drugs and Psychotropic Substances and Precursors thereof §15^1^ [16]). These data consisted of a PB-specific unique person identifier, date of birth, sex, initials, date(s), and county (counties) of misdemeanour(s) residence for each person. Personal communication with PB (T. Riik, The Estonian Police and Border Guard Board) revealed that about 13% of the confiscations in 2015 were related to opioid use. Therefore, for the current analysis, we considered two assumptions: (a) all persons in the PB dataset were PWID and (b) not everyone in the PB dataset was PWID. We analysed data accordingly (see the ‘Statistical analysis’ section).

The Estonian Health Insurance Fund (HIF) provided two sets of data representing two separate ‘captures’: (1) insurance claims related to opioid-related dependence treatment (T40.0–T40.2) (HIF-T) and (2) insurance claims related to opioid-related overdose (F11.0–F11.9) (HIF-F). Both datasets contained the individual’s date of birth, sex, initials, place of residence, and the date of claim; there was no HIF-specific unique code to distinguish persons with the same initials, sex, and date of birth. We assumed that all such insurance claims were related to injecting drug use.

### Linking

Within each of the four datasets, we created a unique identifier (study ID) based on the individual’s date of birth, sex, and initials. We linked datasets covering 2010–2015 by the identifier, taking into account the order of dates of death and other events (insurance claims, misdemeanours). As persons in the DR and PB datasets were uniquely identifiable and distinguishable independently of the study ID, in a case of two or more people having the same study ID, only one of them was linked to the other data sets.

### Statistical analysis

We assessed dependence between four datasets with logistic regression using three datasets for each assessment: one ‘population’ (e.g. DR) and two ‘sampling’ datasets (e.g. PB and HIF-T). Within the ‘population’ dataset, the odds of being captured by one of the ‘sampling’ datasets were compared to the odds of being captured by the other ‘sampling’ dataset; odds ratio (OR) significantly different from 1 suggested dependency between a ‘sampling’ dataset and the ‘population’ dataset.

For each year, we calculated the age of each person as at mid-year and set four indicators denoting presence or absence of the person within four registries in that year; those younger than 15 in a given year were excluded from the analysis of that year. We set the county of residence by the first appearance of the person in any of the registries for that year. Data were aggregated as contingency tables with counts of persons corresponding to each combination of four indicator variables and levels of covariates (age group, sex, county of residence) (Additional file 1).

We used maximum likelihood and Bayesian approaches to estimate the population size. Firstly, to allow comparability with the prevalence estimates from the previous study [8], maximum likelihood Poisson regression models with registry indicators as predictors of the counts of PWID were used. From the model with all main effects and pairwise interactions between the indicators, the interactions were dropped one by one until reaching the lowest Akaike Information Criterion (AIC). This was done separately for each year and for those aged 15–44 as well as for all persons aged 15 years or more. We present point estimates with 95% confidence intervals (CI) in this paper.

Secondly, we used a Bayesian approach described by Overstall et al. [17], allowing us to take into account that possibly a significant proportion of the PB data were non-injecting drug users. Those cells in the contingency tables containing the number of persons captured by PB only were considered left-censored [17], i.e. we assumed that the true numbers of PWID in those cells were lower than or equal to the observed numbers. Generalised hyper-g prior distribution [18] with non-informative parameters *a* = *b* = 10^−3^ was used for Poisson log-linear models fitting the contingency table. The propensity to be included in the registries might be affected by sex, age (15–44 vs 45+) and county of residence (Harju/Ida-Viru/other); therefore, these covariates were included in the models. All hierarchical Poisson models from the set of models with main effects only as minimal model and main effects plus all pairwise interactions as maximal model were fitted, and the results were weighted using Bayesian model averaging as described by Overstall and King [19]. Markov chain Monte-Carlo (MCMC) estimates were compared to data using Bayesian *p* values (based on the chi-squared statistic to describe the discrepancy between the data and the fit [19]). Based on the initial assessment, it was found that the length of 500,000 generally resulted in acceptable mixing and convergence of MCMC chains. We assessed convergence and stationarity of the chains visually and prolonged them if necessary. The first 10% of MCMC iterations were discarded. We present medians of the MCMC estimates with 95% highest posterior density intervals (HPDI).

To compare the Bayesian and maximum likelihood results, we conducted similar Bayesian analysis assuming all persons in the PB dataset to be PWID (PB not censored) and did not adjust capture probability for age, sex, or county of residence. So, in total, there were three types of Bayesian estimates: (1) not accounting for over-coverage of PB and covariates; (2) accounting for over-coverage of PB, but not for covariates; and (3) accounting for over-coverage of PB and covariates (age, sex, county of residence). All Bayesian estimates used the full dataset of PWID at least 15 years old.

We used statistical software R [20] with packages conting [21], coda [22], foreach [23], doParallel [24], and ggplot2 [25] for data preparation, analysis, and presentation.

We obtained mid-year general population size data for PWID prevalence estimates from Statistics Estonia [26].

Estimates of the number of PWID presented in the paper are rounded to the closest hundred. Unrounded estimates are provided in the Additional file 2.

## Results

From 2010 through 2015, there were 721 unique persons in the DR dataset, 8487 in the PB dataset, and 2517 unique study IDs in the HIF datasets (463 in HIF-T and 2202 in HIF-F). Four datasets were positively dependent; odds of being within one dataset were correlated with higher odds of being in the other datasets as well (ORs ranging from 1.25 to 7.19).

**Fig 1:**
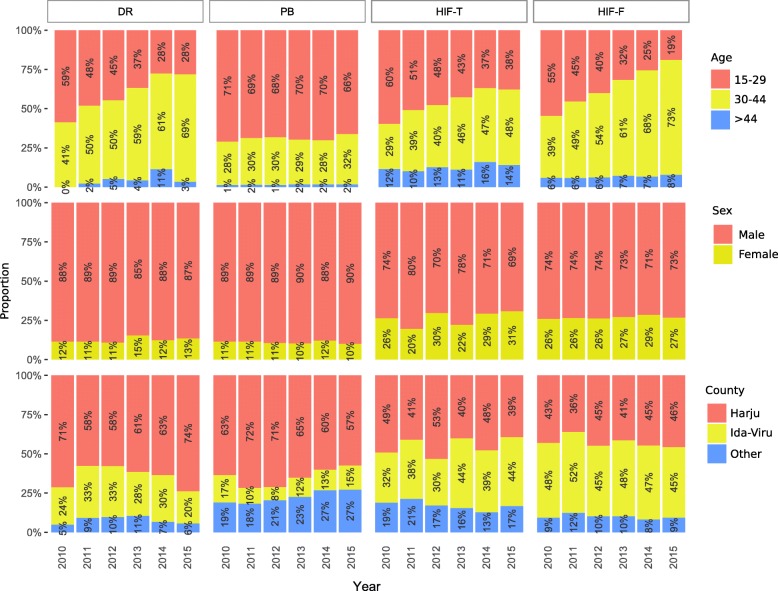
Yearly sample proportions of people in four capture data sets by age, sex, and county. DR, the Estonian Causes of Death Registry; PB, the Estonian Police and Border Guard Board; HIF-T, T40.1–T40.4 persons having insurance claims in the Estonian Health Insurance Fund; HIF-F, F11.0–F11.9 persons having insurance claims in the Estonian Health Insurance Fund

There were 104 opioid-related deaths (DR) in 2010, 131 in 2011, 175 in 2012, 117 in 2013, 105 in 2014, and 89 in 2015. The annual numbers of people detained or arrested due to using or carrying small amounts of drugs (PB) were 1462, 1785, 2174, 2184, 1824, and 2406 for years 2010–2015, respectively. There were 52, 59, 63, 89, 106, and 106 persons who received overdose treatment related to opioids (HIF-T) and 865, 714, 860, 750, 730, and 728 persons who received opioid addiction treatment (HIF-F) in 2010–2015, respectively. Sample distributions of age, sex, and county by years are given in Fig. 1. Excluding PB, the average age increased throughout the years; the proportion of women was higher in HIF-F and HIF-T than in other registries; and the proportion of persons from Ida-Viru County was noticeably smaller in PB than in other registries.

Most of the MCMC chains for the Bayesian estimates had a length of 500,000 (the maximum chain length was to 1500,000 for estimates without covariates for 2010). The Metropolis-Hastings algorithm acceptance rate varied between 30 and 84%; out of 64 Bayesian *p* values, 9 were < 0.05 (4 of them for 2010; 5 *p* values were for estimates not accounting for over-coverage, and the minimum *p* value was 0.0079 for estimates adjusted for covariates for 2014 not accounting for over-coverage).

**Fig 2:**
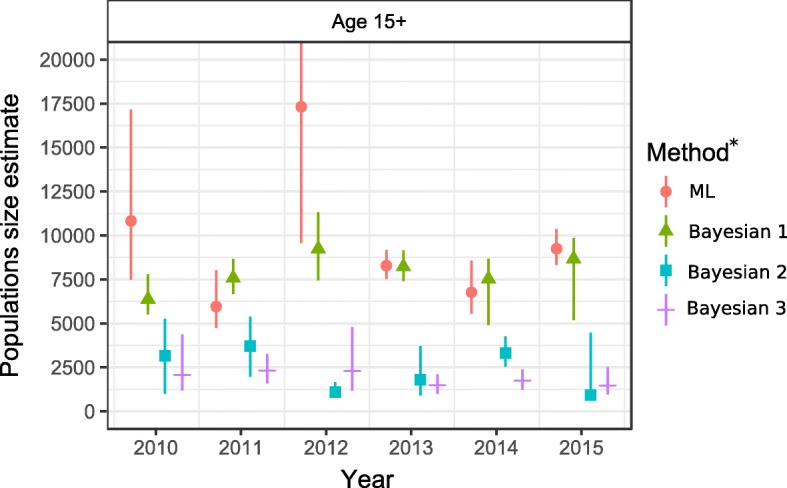
Maximum likelihood estimates with 95% confidence intervals and Bayesian median estimates with 95% highest posterior density intervals. ML, maximum likelihood estimates not accounting for over-coverage of PB and covariates; Bayesian 1, Bayesian estimates not accounting for over-coverage of PB and covariates; Bayesian 2, Bayesian estimates accounting for over-coverage of PB, but not for covariates; Bayesian 3, Bayesian estimates accounting for over-coverage of PB and covariates (age, sex, county of residence)

For the 15–44-year age-group, the maximum likelihood point estimates of the number of PWID in that age group from models with lowest AIC was 13,100 (95% CI 8200–23,600) for 2010; 5500 (95% CI 4400–7400) for 2011; 16,000 (95% CI 8800–31,800) for 2012; 7700 (95% CI 7000–8500) for 2013; 7300 (95% CI 6600–8200) for 2014; and 8600 (95% CI 7700–9700) for 2015 (Additional file 2). When full data of PWIDs aged 15 or more years was used, ML estimates ranged between 6000 and 17,300 (Fig. 2 and Additional file 2).

Ignoring the possibility that PB data included non-injectors, and also ignoring the effect of covariates Bayesian estimates (Bayesian 1) did not differ substantially from ML estimates. Bayesian median estimates for PWID aged 15 years or more varied between 6400 (in 2010, 0.57% prevalence, respectively) and 9200 (in 2012, 0.83% prevalence, respectively) (Fig. 2 and Additional file 2).

Bayesian estimates (Bayesian 2, Bayesian 3) accounting for over-coverage were substantially lower than the ML estimates. When taking censoring into account but still ignoring the covariates (Bayesian 2), median estimates for the total population (aged 15+ years) ranged from 934 PWID/0.08% prevalence in 2012 to 3700 PWID/0.33% prevalence in 2011. Compared to the estimates not taking covariates into account, the estimates taking sex, age (15–44/45+), and county of residence (Harju/Ida-Viru/other) into account and their variability were generally lower; the median estimates for PWID aged 15+ varied between 1500 (2015, prevalence 0.13%) and 2300 (2011, prevalence 0.21%) (Bayesian 3).

**Fig 3:**
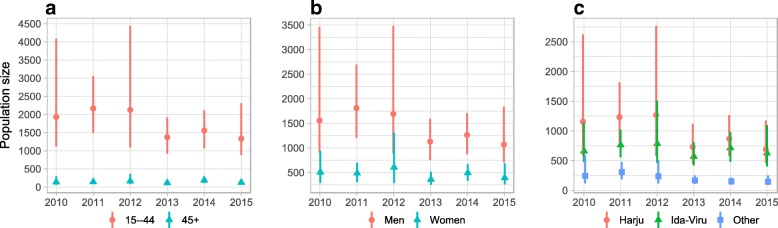
Bayesian estimates of the number of PWID by **a** age, **b** sex, and **c** county of residence (medians and 95% highest posterior density intervals)

In addition, we estimated the sizes of PWID population by sex, age, and county, based on the Bayesian 3 model (accounting for over-coverage of PB and the effect of covariates) (Fig. 3 and Additional file 2). Median estimates for PWID aged 15–44 ranged between 1300 (2015, 91% of PWID, 0.3% of general population in the same age) and 2200 (2011, 94% of PWID, 0.4% of general population in the same age), and for PWID aged over 44 between 100 (2015, 9% of PWID, < 0.1% of general population in the same age) and 200 (2014, 11% of PWID, < 0.1% of general population in the same age). Estimates for the number of men who inject drugs ranged between 1100 (2015, 52% of PWID, 0.2% of men of same age in general population) and 1800 (2011, 79% of PWID, 0.4% of men of same age in general population), and the range for women was from 400 (2013, 24% of PWID, 0.1% of women of same age in general population) to 600 (2012, 26% of PWID, 0.1% of women of same age in general population). The majority of PWID lived in Harju county, with median estimates ranging between 700 (2015, 47% of PWID, 0.2% of Harju county population > 14 years old) and 1300 (2012, 55% of PWID, 0.4% of Harju county population > 14 years old). Median estimates for PWID living in Ida-Viru county ranged between 600 (2013, 39% of PWID, 2.3% of Ida-Viru county population > 14 years old) and 800 (2012, 34% of PWID, 2.9% of Ida-Viru county population > 14 years old).

## Discussion

This paper extends the period for which the national estimates for the numbers of PWID in Estonia are available (from 2005 to 2015).

There was a slight decrease in the Bayesian estimates accounting for over-coverage of PB: the median estimates were > 2000 in years 2010–2012 and < 1800 in years 2013–2015, affirming the trend observed in a previous paper [27]. Most notably, comparing the year 2010 to 2015, the number of PWID has decreased among men (31% vs. 23% among women), in Harju county (40% vs. 5% in Ida-Viru county), and among 15–44-year-olds (31% vs. 7% among over 44-year-olds).

Further, we would like to highlight the importance of accounting for over-coverage in selected data sources. Under the assumption that all people arrested for possessing or using small amounts of drugs were PWID, the Bayesian estimates for the total number of PWID increased from 6200 (5300–7400, or 0.5–0.7% prevalence) in 2010 to 7900 (6700–9300, or 0.6–0.8% prevalence) in 2015. However, the trend was decreasing when the over-coverage was accounted for: from 2100 (1100–4400, or 0.1–0.4% prevalence) in 2010 to 1500 (1000–2500, or 0.1–0.2% prevalence) in 2015.

For the period under observation, there were no significant changes in the spectrum or volume of the harm reduction services in Estonia, nor were there changes in the police practices pertinent to handling drug use-related events. The increasing age of PWID observed in other studies [11, 13] was also reflected in the data used in the current study. As there were no clear patterns in the number of opioid-related deaths during the period, we conclude that the decreasing trend in the estimates of the number of PWID (taking the over-coverage and covariates into account) hints to an underlying true trend of decreasing population of PWID.

Patterns of drug use may change through time as well as the composition of the population of PWID itself. Although in the previous study it was assumed that the vast majority (80–90%) of drug users arrested by police were injecting drug users [8], our data did not support this. The studies conducted in Tallinn have shown that the population of PWID is getting older [11, 13] which was also clearly reflected in datasets from the death registry and the health insurance fund, but not in data on police arrests in which only a minority (close to 10%) was related to opioids in 2015. Hence, it can be assumed that police data is left-censored, the true number of PWID captured by the police dataset is less than observed, and there is over-coverage present.

There might exist similar problems related to case definition in the other three sources as well. While most PWID use fentanyl or other opioids (76% via injecting or other modes during the previous 6 months in Tallinn in 2016 (A. Uusküla, unpublished data)), a significant proportion uses other stimulants only (mainly amphetamine, 24% in Tallinn in 2016 (A. Uusküla, unpublished data)). Hence, the opioid-related queries from the death registry and health insurance fund did not cover such PWID and corresponding numbers are right-censored (true numbers of drug-related deaths, overdose, and dependence treatment among PWID are probably somewhat higher than observed), and the number of all PWID (including amphetamine-injecting PWID) might be underestimated. Furthermore, some PWID are not covered by the Estonian Health Insurance Fund (e.g. about 8% of PWID in Kohtla-Järve in 2016 [28]), and although receiving emergency care in case of overdose, they do not receive drug dependence treatment, excluding methadone substitution which is paid for by the Ministry of Social Affairs, but not by the Estonian Health Insurance Fund. Additionally, the three datasets (DR, HIF-T, HIF-F) might also have captured non-injecting drug users and it is difficult to classify a person as an injecting or a non-injecting drug user, based solely on the variables in these datasets. For example, among opioid-related deaths, there might have been some persons who used fentanyl by sniffing. According to a report [28], about 84% of persons seeking drug treatment in 2010 used fentanyl as their main drug, and more than 90% did so by injecting. Hence, among persons receiving drug treatment, there are non-injecting fentanyl users; the overwhelming majority of fentanyl users probably are injecting drug users. Unfortunately, Estonian Causes of Death Registry do not record the mode of intake of the substances. However, according to Paabo [15], about 85% of overdose deaths in 9 months in 2012 were related to fentanyl use. As a large part of fentanyl users seeking drug treatment were PWID, we reason by analogy that the majority of such fentanyl-related deaths were among injecting drug users.

Conclusively, as there are no variables in the datasets to separate non-injecting drug users from PWID, there probably are both under- and over-coverage present in the datasets. Notwithstanding, as the demographics in data from death registry and health insurance fund correspond to the cross-sectional studies conducted among PWID (e.g. ageing of the population), we hypothesise that the total effect of over- and under-coverage of death registry and health insurance fund datasets introduces less bias than the one introduced by police data.

Additionally, we acknowledge that all of the four datasets capture probably mostly those PWID who have high-risk behaviour (e.g. resulting in overdoses) and PWID with low death or overdose risk are under-represented in these data. Therefore, the estimates for the total PWID population size can be somewhat higher. To reduce the potential bias, we used modelling including covariates [29]. We estimated the number of PWID by age group, sex, and county of residence, which helped us to compare the results to those seen in respondent-driven-sampling studies conducted in Tallinn (the capital city) and Kohtla-Järve (fifth largest city in the country). The proportion of women among PWID has been estimated around 23% in 2016 in Tallinn [11] and around 26% in 2012 in Kohtla-Järve [12], and the proportion of PWID older than 44 years was estimated at 6% and 1% in those studies, respectively (Uusküla, unpublished data). In our study, when not taking over-coverage of police data into account, the Bayesian estimate of the proportion of female PWID in 2015 was 14% while modelling the over-coverage the estimate for the proportion of women (27%) was in better concordance with respondent-driven-sampling studies. Corresponding estimates of proportions of PWID older than 44 years in 2015 were 7% (maximum likelihood) and 9% (Bayesian).

Further, to investigate the potential limitations of our analysis, we will address the fitness of our data to the analytic approach selected. There are four classical assumptions in capture-recapture studies [30]. Two of them, the independence of captures and homogeneity of capture probability, can be relaxed by including interaction terms between the registries and between the covariates to the log-linear regression models. This was done in our study. We had data on three covariates (age, sex, county of residence) which probably correlate strongly with behaviour and, therefore, with capture probability. We did not have data on socio-economic factors such as levels of education and income; hence, there might be some bias caused by the heterogeneity associated with those factors. For example, if PWID with higher education are less prone to overdose, die, or be arrested, then this would result in bias decreasing the estimates. The direction and extent of such bias is not estimable using the data of this study. However, we noted that the results taking account age, sex, and county of residence were mostly (but not always) lower than the results not taking these factors into account, and year-on-year variability of the estimates was lower.

Another classical requirement of the capture-recapture method is the closedness of the population. In our study, the average number of deaths registered per year was 120, so this requirement is not fully met. However, we used 1-year periods during which it is reasonable to assume that the change of population due to migration and new people starting injecting is relatively small.

The fourth assumption in capture-recapture studies is a perfect linkage of the captures. The identifying variable used in this study (study ID) was based on sex, date of birth, and the initials of the persons; there were no missing IDs. Two persons had the same study ID in the death registry data, but due to the nature of the registry, they were different persons. The police dataset contained additional IDs provided by the police, making different persons with the same study ID identifiable. Among all study IDs (*n* = 8491) in the police dataset, there were only 30 (0.04%) which corresponded to more than one person. As the model estimates are based on the cell counts in frequency tables, it does not matter whether one or the other of the two persons with the same study ID gets linked to the other registry, it is only important that they both do not get linked simultaneously. However, data provided by the health insurance fund did not have additional internal ID variable and it was not possible to find out how many people did have the same study ID. We believe that the proportion of such study IDs is similar to those in police data. Hence, although it is probable that different people with the same study ID in the health insurance fund data got linked to a single person in police or death registry data, the resulting bias is probably negligible.

## Conclusion

Over-coverage of a registry can have an important impact on estimates of the number of PWID. Bayesian estimates accounting for this over-coverage may provide better estimates of the target population size.

## Additional files

Additional file 1: Capture frequencies by gender, age group, county of residence, year, and registries. (XLSX 54 kb)

Additional file 2: Bayesian and maximum likelihood estimates of the total number of PWID for years 2010-2015. (XLSX 20 kb)

## Abbreviations

AIC: Akaike Information Criterion
CI: Confidence interval
DR: The Estonian Causes of Death Registry
HIF: The Estonian Health Insurance Fund
HPDI: Highest posterior density intervals
ICD: The International Classification of Diseases
MCMC: Markov chain Monte-Carlo
ML: Maximum likelihood
OR: Odds ratio
PB: The Estonian Police and Border Guard Board
PWID: People who inject drugs
UNODC: The United Nations Office on Drugs and Crime
